# Endophytic bacterial communities are associated with leaf mimicry in the vine *Boquila trifoliolata*

**DOI:** 10.1038/s41598-021-02229-8

**Published:** 2021-11-22

**Authors:** Ernesto Gianoli, Marcia González-Teuber, Claudia Vilo, María J. Guevara-Araya, Víctor M. Escobedo

**Affiliations:** 1 Departamento de Biología, Universidad de La Serena, La Serena, Chile; 2 Departamento de Química Ambiental, Universidad Católica de la Santísima Concepción, Concepción, Chile; 3 Departamento Biomédico, Facultad de Ciencias de la Salud, y Centro de Biotecnología y Bioingeniería, Universidad de Antofagasta, Antofagasta, Chile; 4 Instituto de Ciencias Biológicas, Universidad de Talca, Talca, Chile

**Keywords:** Ecology, Evolution

## Abstract

The mechanisms behind the unique capacity of the vine *Boquila trifoliolata* to mimic the leaves of several tree species remain unknown. A hypothesis in the original leaf mimicry report considered that microbial vectors from trees could carry genes or epigenetic factors that would alter the expression of leaf traits in *Boquila*. Here we evaluated whether leaf endophytic bacterial communities are associated with the mimicry pattern. Using 16S rRNA gene sequencing, we compared the endophytic bacterial communities in three groups of leaves collected in a temperate rainforest: (1) leaves from the model tree *Rhaphithamnus spinosus* (RS), (2) *Boquila* leaves mimicking the tree leaves (BR), and (3) *Boquila* leaves from the same individual vine but not mimicking the tree leaves (BT). We hypothesized that bacterial communities would be more similar in the BR–RS comparison than in the BT–RS comparison. We found significant differences in the endophytic bacterial communities among the three groups, verifying the hypothesis. Whereas non-mimetic *Boquila* leaves and tree leaves (BT–RS) showed clearly different bacterial communities, mimetic *Boquila* leaves and tree leaves (BR–RS) showed an overlap concerning their bacterial communities. The role of bacteria in this unique case of leaf mimicry should be studied further.

## Introduction

Mimicry phenomena, whereby one species imitates another and, in so doing, gains fitness benefits, have long attracted ecological and evolutionary research, but cases in plants are not numerous^1–3^. A remarkable example of mimicry in plants is found in Australian mistletoes, whose leaves mimic those of their specific host trees^4–6^, but see^7^. Arguably the most striking case of mimicry in plants is the reported leaf mimicry by *Boquila trifoliolata* (Lardizabalaceae)^8^, a twining vine endemic to the temperate rainforest of southern South America^9^. *Boquila* is able to mimic the leaves of over a dozen tree species when growing onto them or in close proximity^8,10^. Moreover, an individual *Boquila* plant associated with two different tree species can mimic both of them^8^. Leaf mimicry by *Boquila* has been characterized in terms of leaf size, shape, colour, orientation, petiole length, and leaf tip spininess^8,10^. Field evidence of leaf morphology and herbivore damage in (i) unsupported vines, (ii) climbing vines closely associated with tree foliage, and (iii) vines climbing onto leafless trunks^8^, strongly suggests that *Boquila* gains protection against herbivory not only by climbing, and hence avoiding herbivores in the ground^11,12^, but also by climbing trees whose leaves are actually mimicked. Thus, the palatable but mimetic *Boquila* associated with less palatable tree species would receive less damage by visually-oriented herbivores^8^. Leaf mimicry by *Boquila* has puzzled the scientific community since it was first reported, and the mechanisms underlying this phenomenon are yet to be identified.

Whereas plausible selective agents responsible for mimicry cases may be inferred after a thorough ecological knowledge of the study system^1–3^, the elucidation of the physiological or molecular mechanisms behind these phenomena requires greater research efforts. Perhaps the closest case to leaf mimicry by *Boquila* is that of Australian mistletoes, a system where the roles of herbivores^4–6^ and seed-dispersing birds^13,14^ as selective agents have met supporting evidence. However, the proposed explanatory mechanism for mistletoe mimicry, which considers the sharing of morphogenetic hormones such as cytokinins^15,16^, is still under debate^14,17^. The facts that mimetic Australian mistletoes are hemiparasites physiologically connected to their host trees^6,17^, and that they often show associations with a single or a few host species^4,17^, make the identification of the mechanisms underlying leaf mimicry a seemingly reachable goal. In the case of *Boquila*, the link between herbivore damage and leaf mimicry has been established^8^. However, deciphering the mechanism behind the exceptional capacity of leaf mimicry in *Boquila* is indeed a challenging, complex task.

Two hypothetical explanatory mechanisms for leaf mimicry in *Boquila* were outlined in the original study^8^: (i) volatile compounds emitted by trees could modulate gene expression in *Boquila*, and (ii) microbial vectors could carry genes or epigenetic factors from trees to *Boquila* that would alter the expression of leaf traits. The first hypothesis could explain the observed leaf mimicry without direct contact and is generally supported by the fact that volatile plant communication is widespread and multi-purpose^18^. Nonetheless, to our knowledge, there is no documented evidence of changes in leaf shape elicited by volatiles and, more importantly, known volatile-mediated responses in receiver plants are rather general^18–21^, while leaf mimicry in *Boquila* is highly specific. The second hypothesis, the horizontal gene transfer (HGT) hypothesis, has been deemed implausible^22–24^. However, evidence from other study systems suggests that the HGT hypothesis is not too speculative. First, HGT has been demonstrated for a number of plant species^25–29^, including transposable elements^30,31^. Second, HGT may have an adaptive value in natural populations: in a grass species, a transgene acquired from a distantly related grass contributes to local adaptation to microenvironmental variation^32^. Third, vector-mediated HGT has been reported for several plant species. Published cases involve transposons as well as microbial vectors such as fungi, bacteria and viruses^29^. Fourth, bacteria themselves may affect epigenetic factors, ultimately influencing gene expression^33–35^.

**Figure 1:**
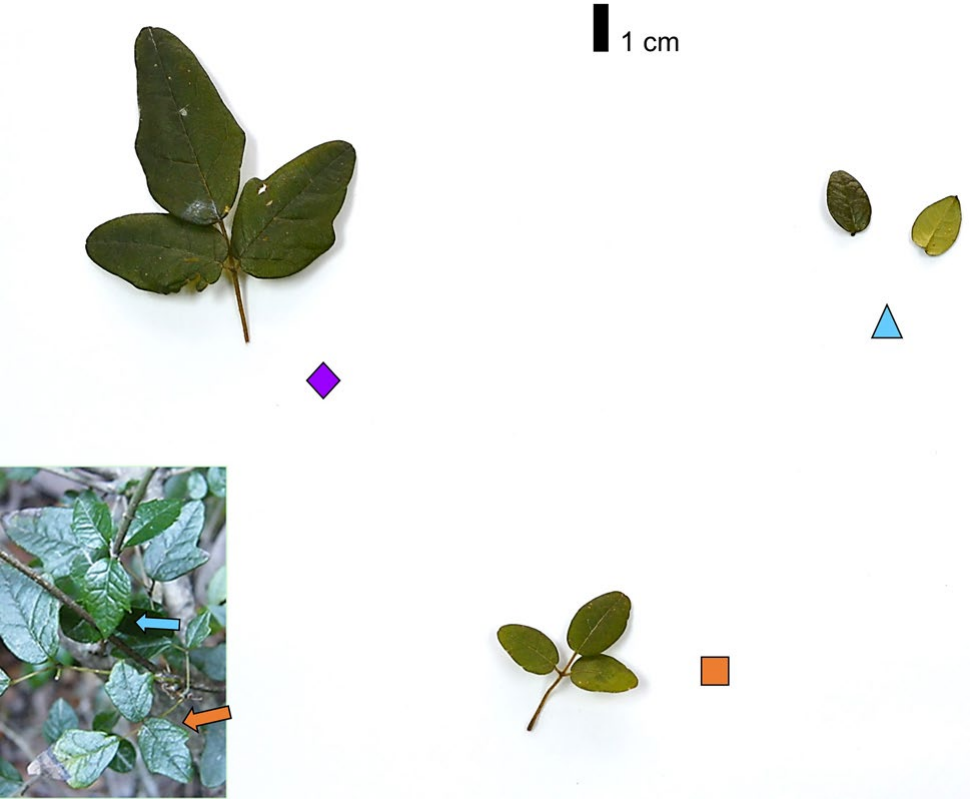
One of the five sets of leaf samples collected in the field in associations between the model tree *Rhaphithamnus spinosus* and the vine *Boquila trifoliolata*. RS = two leaves from *R. spinosus* [sky blue triangle], BR = a single *Boquila* leaf—composed of three leaflets—mimicking the tree leaves [orange square], and BT = a single *Boquila* leaf from the same individual vine but not mimicking the tree leaves [purple rhombus]. Inset: field picture showing leaf mimicry of *R*. *spinosus* [sky blue arrow] by *Boquila* [orange arrow] (photo credit: E. Gianoli). Note that leaf mimicry is accomplished for both ovate leaves (study samples) and cordate-lobed leaves (inset) of the tree. For other cases of *Boquila* mimicking *R*. *spinosus* see^8,10^.

As a first step to unravel the mechanisms behind leaf mimicry in *Boquila*, and in the context of the HGT hypothesis, here we addressed whether leaf endophytic bacteria are associated with the mimicry pattern. Leaf endophytic bacterial communities have been shown to play significant roles in plant metabolism and ecological interactions^36,37^. Using 16S rRNA gene sequencing, we determined the taxonomic richness and composition of the leaf endophytic bacterial communities in a common *Boquila*-tree association at a temperate rainforest in southern Chile. Specifically, we compared the endophytic bacterial communities in three groups of field-collected leaf samples: RS = leaves from the model tree species, *Rhaphithamnus spinosus* (Verbenaceae), BR = *Boquila* leaves mimicking the tree leaves, and BT = *Boquila* leaves from the same individual vine but not mimicking the tree leaves (Fig. 1). We hypothesized that, if bacterial vectors are involved in the leaf mimicry phenomenon, the bacterial community from group BR (mimetic *Boquila*) would be more similar to that from group RS (model tree) than the bacterial community from group BT (non-mimetic *Boquila*).

## Results

**Figure 2:**
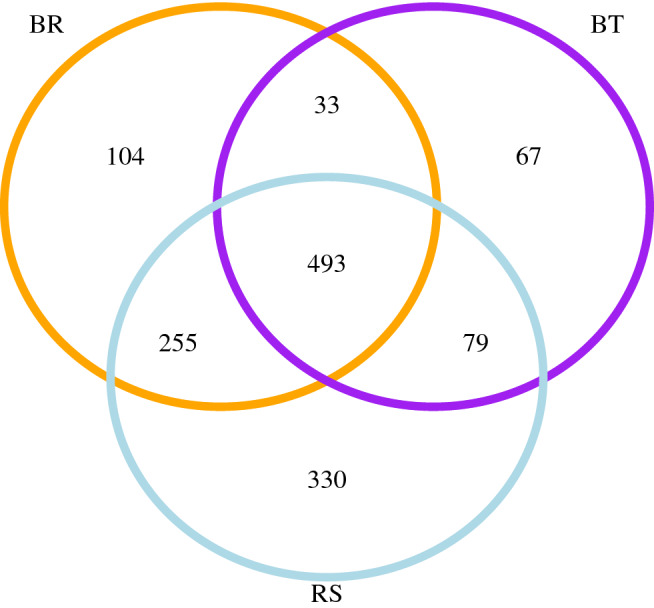
Venn diagram showing the number of unique and shared bacterial OTUs among field-collected leaf samples from the association between the model tree *Rhaphithamnus spinosus* and the mimetic vine *Boquila trifoliolata*. Groups: RS = leaves from *R. spinosus* [sky blue], BR = *Boquila* leaves mimicking the tree leaves [orange], and BT = *Boquila* leaves from the same individual vine but not mimicking the tree leaves [purple].

A total of 45 bacterial phyla were detected across leaf samples, with Proteobacteria, Thermotogae and Actinobacteria comprising over 75% of taxa (Supplementary Figure S1). Overall, we identified 1571 bacterial OTUs (Operational Taxonomic Units). The average number of bacterial OTUs differed among the three groups of leaf samples (*F*_2,8_ = 11.59, *P* = 0.004; one-way ANOVA). ANOVA assumptions of data normality and homoscedasticity were met. Tukey HSD tests showed that the number of bacterial OTUs was significantly lower in BT (269.4 ± 38.5, mean ± SE) compared to both BR (435.6 ± 26.4) and RS (589.2 ± 59.2), whereas no significant differences were found between BR and RS in the number of bacterial OTUs. A Venn diagram shows that BT and BR shared 33 unique OTUs, whereas BT and RS shared 79 unique OTUs (Fig. 2). Remarkably, BR and RS (i.e., mimetic *Boquila* and the model tree) shared 255 unique OTUs (Fig. 2).

**Figure 3:**
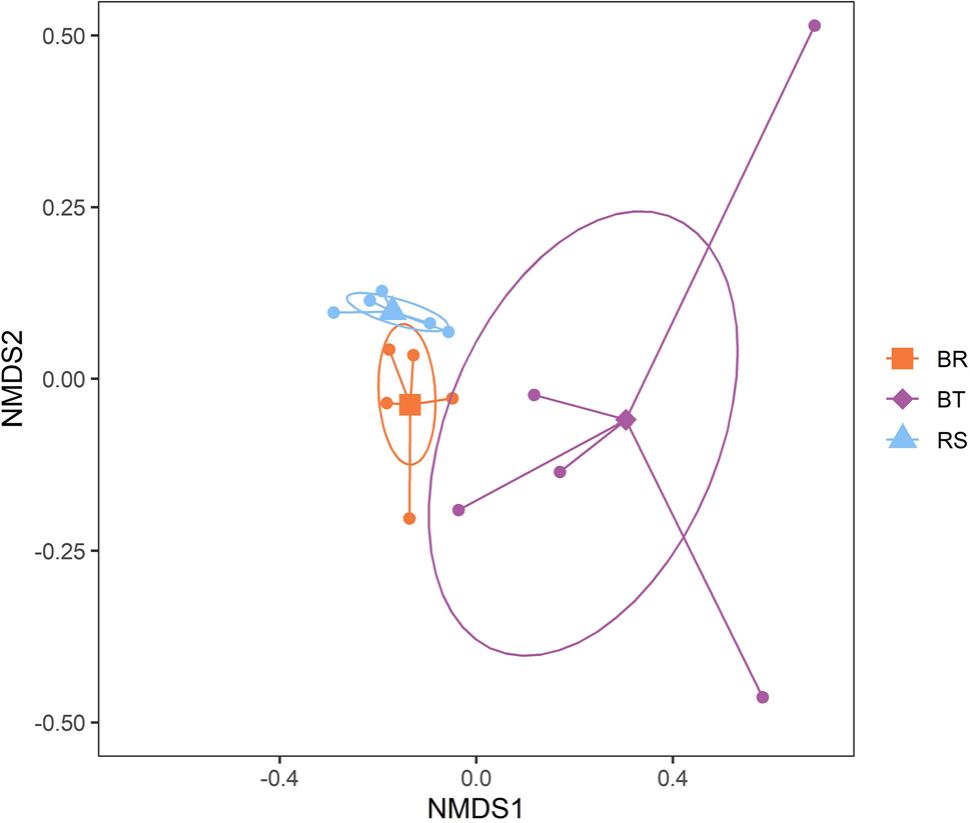
Compared community composition of endophytic bacterial communities in field-collected leaf samples from the association between the model tree *Rhaphithamnus spinosus* and the mimetic vine *Boquila trifoliolata*. Groups: RS = leaves from *R. spinosus* [centroid: sky blue triangle], BR = *Boquila* leaves mimicking the tree leaves [centroid: orange square], and BT = *Boquila* leaves from the same individual vine but not mimicking the tree leaves [centroid: purple rhombus]. Patterns are based on a two-dimensional non-metric multidimensional scaling (NMDS) analysis (final stress = 0.063). Standard error ellipses show 95% confidence areas.

The PERMANOVA results indicate that there were significant differences in the endophytic bacterial communities among the three groups of leaf samples (Table 1). The NMDS patterns, based on Bray–Curtis dissimilarity, indicate that—despite the small number of replicates—our hypothesis was verified, i.e., the bacterial community from mimetic *Boquila* (BR) was more similar to that from the model tree (RS) than the bacterial community from non-mimetic *Boquila* (BT) (Fig. 3). Whereas non-mimetic *Boquila* leaves and tree leaves (RS–BT) showed different endophytic bacterial communities, mimetic *Boquila* leaves and tree leaves (RS–BR) showed a slight overlap in the 95% confidence areas concerning their endophytic bacterial communities (Fig. 3). Accordingly, the distance between the centroids of RS and BT was 0.50, while the distance between the centroids of RS and BR was 0.14 (Fig. 3). The distance between the centroids of BT and BR was 0.44 (Fig. 3). There was concordance between observed interobject distances and those predicted from the dissimilarities (final stress = 0.063). Interestingly, the dispersion of points was much greater in the potentially multi-phenotype *Boquila* (BT) than in the model tree (RS) or in *Boquila* mimicking the tree (BR) (Fig. 3).

**Table 1:** Permutational multivariate analysis of variance (PERMANOVA) of endophytic bacterial communities (Operational Taxonomic Units—OTUs, presence/absence data) in three groups of samples: leaves from the model tree species *Rhaphithamnus spinosus*, leaves from the twining vine *Boquila trifoliolata* actually mimicking the tree leaves, and *Boquila* leaves from the same individual vine but not mimicking the tree leaves (*n* = 5 replicates per group).

	*df*	*SS*	*MS*	Pseudo-*F*	*R*^2^	*P*
Group	2	0.457	0.229	2.104	0.26	0.005
Residuals	12	1.303	0.109		0.74	
Total	14	1.760

## Discussion

We found that mimetic *Boquila* were closer to the model tree *Rhaphithamnus spinosus* than non-mimetic *Boquila* in terms of the composition of endophytic bacterial communities, with over three-times more shared unique OTUs and less than one-third the distance between centroids in the NMDS analysis. Our results suggest the involvement of bacterial agents in leaf mimicry by *Boquila*, yet we are still far from proving the HGT hypothesis. Thus, here we validate—and promote further research on—the role of bacteria in this unique case of leaf mimicry. Although there were 255 bacterial OTUs exclusively shared by mimetic *Boquila* and the model tree, and it could be tempting to delve further into this group, here we refrain from attempting to identify particular bacterial taxa that presumably could play a role in the leaf mimicry phenomenon. Such a specific question should be tackled with a different experimental approach, e.g., sequentially excluding particular bacterial taxa and measuring the expression of leaf traits. More importantly, our hypothesis is that bacteria could be just the vectors carrying genetic or epigenetic factors from the tree to the vine. Thus, it would be of little use for the purpose of testing this hypothesis to search in the literature for reported functional roles of shared bacterial taxa (ideally, linked to leaf traits). Despite its preliminary nature, this study has two main strengths. First, it is based on field-collected samples rather than on greenhouse-grown plants, thus lending ecological realism to the outcome. Second, it has a sound experimental design, which took advantage of the fact that a single *Boquila* individual vine can have both mimetic and non-mimetic leaves. Therefore, when comparing mimetic and non-mimetic leaves in their similarity to leaves of the companion tree *Rhaphithamnus spinosus* regarding endophytic bacterial communities, we could keep constant the vine genotype, the environment, and the model.

HGT cases in plants often involve parasitic plants and their hosts^26,27,29^, which is likely a consequence of their intimate and long-standing contact. Furthermore, known examples of HGT between plants are discrete events that occurred once—or a few times—in evolutionary history^26,29^. These spatial and temporal features of HGT in plants pose significant challenges to the HGT hypothesis in *Boquila*. On the one hand, to account for leaf mimicry in *Boquila* we need, regarding spatial aspects, a mechanism that can be effective without contact between plants, and this is why we considered a microbial airborne vector. On the other hand, regarding temporal aspects, we need a mechanism similar to the above described “historical” HGT, but operating at an ecological time-scale, and this is why we considered potential epigenetic roles of such microbial vectors.

There are several cases of crop mimicry in weeds driven by unintentional selection by farmers, also known as “Vavilovian mimicry”^3,38,39^. For instance, mimetic populations of the weed *Echinochloa crus-galli* and cultivated rice are indistinguishable at the seedling stage, particularly sharing an upright habit of both tillers and leaves^38,40^. In a genomic study comparing mimetic and non-mimetic populations of *E. crus-galli* in rice paddies, Ye et al.^40^ reported that genomic regions harbouring 87 putative plant architecture-related genes were under selection during the differentiation between mimetic and non-mimetic populations, which occurred ≈ 1000 years ago. This study illustrates that, even for a rather simple and widely known mimicry case, elucidation of the underlying mechanisms is a complex task. Therefore, concerning the mechanisms behind mimicry capacities of *Boquila*, we envision a long road ahead of us.

We need to explain not only how *Boquila* is able to mimic over a dozen species in terms of leaf shape and size, even without direct contact, or how a single individual vine can mimic two different tree species^8^. We also need to elucidate how this vine can develop a small spine at the leaf tip when twining around—or being close to— species with such mucronate leaves, which include *Luma apiculata*^8^, *Cissus striata*^10^, and *Rhaphithamnus spinosus* (Gianoli, personal observations: a video footage showing this feature is included in the Supplementary Video S2); importantly, the botanical description of *Boquila* does not include spiny leaf tips^41^. Moreover, concerning the temporal axis of the mimicry phenomenon, and unlike the model-mimic associations lasting for centuries or millennia, we have detected that trailing *Boquila* vines are able to mimic the exotic herb *Ranunculus repens*^10^, which was introduced in the study area a few decades ago^42^. A comprehensive research programme aiming to test the HGT hypothesis for leaf mimicry in *Boquila* will likely include genetic, metagenomic, transcriptomic, proteomic, metabolomic and epigenetic studies, tied to both field and greenhouse experiments. We suggest that such a research programme would eventually crack the code of this amazing plant, and beyond leaf mimicry, help further our understanding of plant phenotypes in general.

## Methods

### Study system

The study was carried out at Anticura, Puyehue National Park, southern Chile (40°39′S, 72°11′W; 350–400 m). In this cold temperate rainforest^43^ the dominant trees are broadleaf evergreen species and woody vines are fairly abundant^9,44,45^. The main herbivores are slugs, snails, weevils and leaf beetles^46,47^. The woody vine *Boquila trifoliolata* (Lardizabalaceae, a monotypic genus) is distributed along the whole light gradient in the mature forest^9^. This twining vine has slender stems when young, and leaves are composed of three pulvinated leaflets^41^. The central leaflet is slightly larger than the lateral ones, and leaflets show significant variation in size (10–100 mm) and shape: ovate-elliptical, oblong-elliptical or obovate; leaf tips are lobulated or emarginated^8,41^. The small tree *Rhaphithamnus spinosus* (Verbenaceae), endemic to the temperate rainforest of southern South America^48^, is commonly found in advanced regeneration stands^49^, but is distributed across the entire light gradient^44^. It is armed with thorns on leaf axils^48^ and has simple, opposite leaves (7–35 mm long, 5–25 mm wide) with ovate or cordate-lobed blades and spiny tips^50^.

### Field sampling

We located five adult individuals of *R*. *spinosus* (height range: 100–170 cm) climbed by *Boquila* vines. Distance between individual trees was 50–900 m. In each of those five tree-vine associations we collected three groups of samples: RS = two leaves from *R. spinosus*, BR = a single *Boquila* leaf (composed of three leaflets) mimicking the tree leaves, and BT = a single *Boquila* leaf from the same individual vine but not mimicking the tree leaves. In order to carry out a proper comparison, in all cases the mimetic and non-mimetic *Boquila* leaves were very close (< 60 cm) and their respective distances to tree leaves were nearly the same (video footage included in the Supplementary Video S1). Collected leaves were placed in paper bags with silica gel within a cooler with ice packs, stored at 4 °C and transported to the lab within 48 h for DNA extraction. This study complies with local and national regulations concerning research and field studies on plants in protected areas. Permission for research and collection of plant material was granted by CONAF (Corporación Nacional Forestal) permit No. 012/2018 to EG. Plant species were identified by the first author, who has over ten years of experience working in the study system.

### DNA extraction, amplification, and sequencing

For DNA extraction, leaves were sterilized with washes of ethanol (70%), sodium hypochlorite (1%) and water. The success of surface sterilization was confirmed by the absence of any microorganism growing on PDA (potato-dextrose-agar) (Phyto Technology Laboratories) plates from the plating of last washing water. Genomic DNA was extracted from 100 mg dry leaf material using a CTAB-based method^51^. DNA samples were amplified by PCR using the primers 515F (5′-GTGCCAGCMGCCGCGGTAA-3′) and 806R (5′-GGACTACHVGGGTWTCTAAT-3′). Primers 515F-806R target the V4 region of the 16 SSU rRNA gene. Since sequencing the V4 region from plant tissues can lead to host-derived plastid and mitochondrial sequences^52^, chloroplast PNA (5′-GGCTCAACCCTGGACAG-3′) and mitochondrial PNA 5′-GGCAAGTGTTCTTCGGA-3′) were used in order to block the amplification of plastid sequences. PCR reactions were carried out in 50 μl final volume containing 1 × SapphireAmp Fast PCR Master Mix (Clontech), 300 nM each primer, 1.25 μM each PNA, 100 ng DNA template and DNA-free water. PCRs were performed in a Techne TC-5000 Thermal Cycler (Fisher Scientific) with the following program: 94 °C for 1 min, 34 cycles of denaturation at 95 °C for 15 s, annealing at 78 °C for 10 s and primer extension at 60 °C for 30 s, and a final extension at 72 °C for 30 s. The amplified products were checked in 1% w/v agarose gels. PCRs were carried out in a PCR laminar flow cabinet in order to prevent cross-contamination during reagent preparation. Amplicons were sequenced on Illumina-MiSeq platform at Macrogen Inc. (Seoul) according to the manufacturer's instructions. Before sequencing, samples were normalized based on Picogreen concentrations (Macrogen Inc.).

The Illumina MiSeq Platform was used to sequence the amplified V4 region of the 16S rRNA gene from metagenomic DNA samples. The reads obtained were paired-ended with a read length average of 300 bp. The TrimGalore wrapper application [http://www.bioinformatics.babraham.ac.uk/projects/trim_galore/] was used to remove adapters and low-quality sequences. The sequencing data was analysed using Mothur software (version 1.38.1)^53^ with the default options, unless otherwise stated. Reads shorter than 200 bp were discarded. Reads were denoised using the “pre.cluster” command in Mothur platform to remove sequences that were likely due to errors and assemble reads that differed only by 2 bp. Chimeric sequences were identified and removed, and the remaining sequences classified against the SILVA database^54^ using the RDP Classifier program^55^. The table of OTUs obtained from all high-quality sequences was defined at 97% similarity level. Customized perl scripts were used to create OTUs and taxonomy tables.

### Statistical analyses

The total number of OTUs was compared among the three groups of leaf samples with a one-way ANOVA (*n* = 5 individuals per group, with each tree-vine association considered a block), followed by a post-hoc Tukey HSD test. A Venn diagram was constructed using the “VennDiagram” package in R; OTUs present in a single replicate were considered as present for the whole group. We tested for differences in the community structure of endophytic bacteria among the three groups using non-parametric Permutational Multivariate Analysis of Variance (PERMANOVA). PERMANOVA was performed through the R-function “adonis()” from the *vegan* package on a matrix based on Bray–Curtis dissimilarity from the presence/absence data, taking blocks (= five tree-vine associations) into account using the “strata” argument. We also implemented a two-dimensional non-metric multidimensional scaling (NMDS) based on Bray–Curtis dissimilarity to visualize community structure changes^56^.

## Supplementary Information

Supplementary Information.

Supplementary Figure S1.

Supplementary Legends.

Supplementary Video S1.

Supplementary Video S2.

## Data Availability

The dataset supporting this article is available as part of the electronic supplementary information. Sequences were deposited in the sequence read archive (SRA) of the National Center for Biotechnology Information (NCBI; BioProject PRJNA479681).
