# Supplementary figures and images for: Endophytic bacterial communities are associated with leaf mimicry in the vine Boquila trifoliolata
Source: Sci Rep. 2021 Nov 22;11:22673. doi: 10.1038/s41598-021-02229-8 (PMC8608808; doi:10.1038/s41598-021-02229-8)

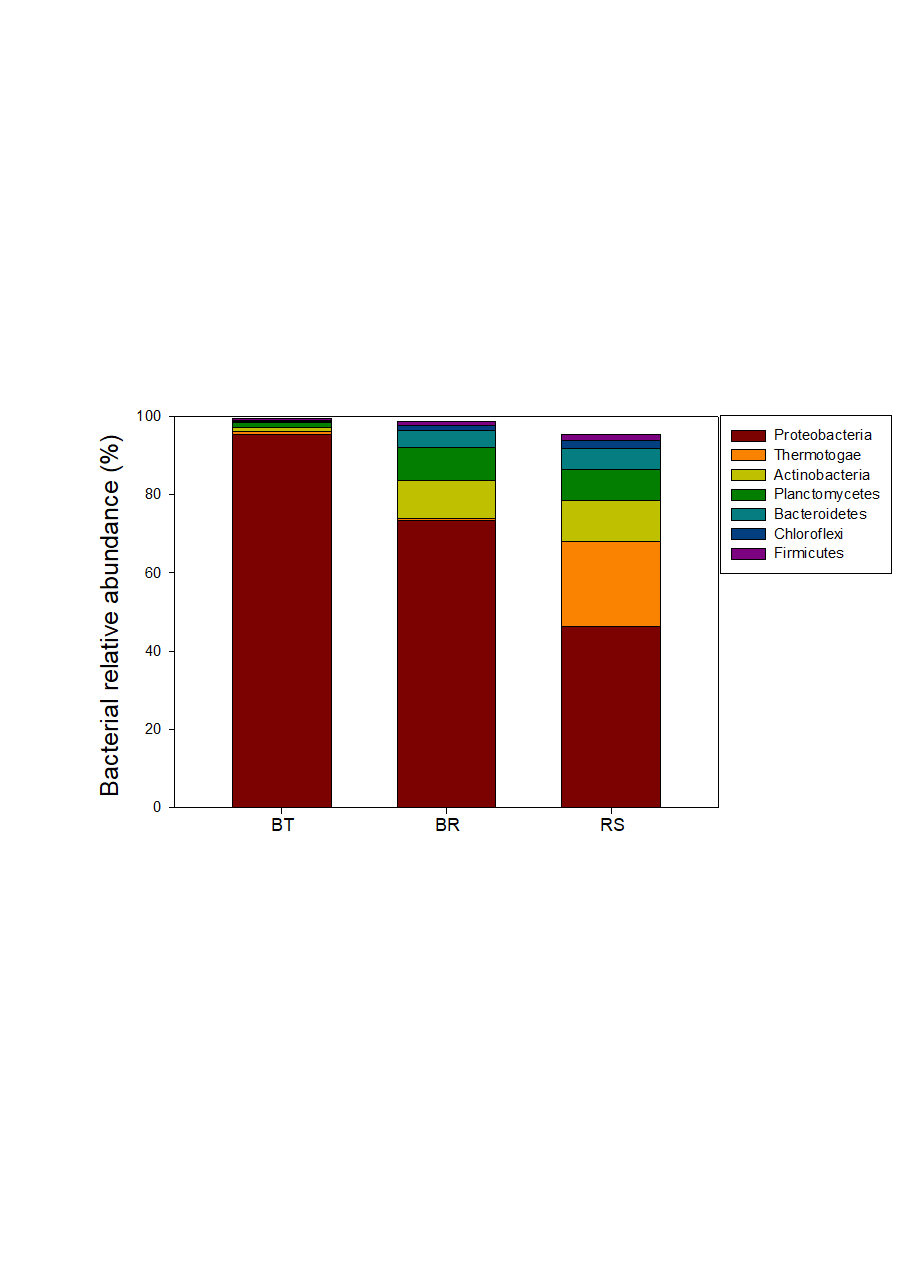

Supplement: Supplementary file 2 — Supplementary Figure S1. [file 41598_2021_2229_MOESM2_ESM.jpg]
