# Supplementary material for: Endophytic bacterial communities are associated with leaf mimicry in the vine Boquila trifoliolata
Source: Sci Rep. 2021 Nov 22;11:22673. doi: 10.1038/s41598-021-02229-8 (PMC8608808; doi:10.1038/s41598-021-02229-8)
Supplement: Supplementary file 3 — Supplementary Legends. [file 41598_2021_2229_MOESM3_ESM.docx]

**Supplementary Information**

**Figure S1.** Relative abundance of bacterial endophyte phyla for BT, BR and RS leaf samples (*n* = 5 individuals). Only phyla with relative abundances above 1% are shown.

**Video S1.** Footage showing mimetic and non-mimetic *Boquila* leaves from the same individual vine associated with the model tree *Rhaphithamnus spinosus*. Sequence: model tree – mimetic *Boquila* – non-mimetic *Boquila*.

**Video S2.** Footage showing the spiny leaf tip developed in *Boquila* leaves (red arrows) when associated with the model tree, which naturally shows spiny tips (blue arrow).
